# Supplementary material for: Accurate auto-labeling of chest X-ray images based on quantitative similarity to an explainable AI model
Source: Nat Commun. 2022 Apr 6;13:1867. doi: 10.1038/s41467-022-29437-8 (PMC8986787; doi:10.1038/s41467-022-29437-8)
Supplement: Supplementary file 1 — Supplementary Information [file 41467_2022_29437_MOESM1_ESM.pdf]

Supplementary Information

Supplementary Table 1 | Detailed information about our institutional datasets. We gathered examinations for both AP and PA view positions, and automatically generated three labels (positive, negative, ignore) by our natural language processing (NLP) tool which had been developed to convert terms used in radiology reports into the labels. This tool is a rule-based approach that has a dictionary of radiology terms, based on Radex Lexicon [1], and a mapping table of 20 pathological labels and the key terms. Label generation of the NLP, using NLTK [2] and Spacy [3], employs two-staged processing for each examination: The first stage extracts radiology terms from a radiology report of the examination using the dictionary while considering both negation and double negation. In the second stage, the NLP determines labels from the terms by a mapping rule defining key terms for 20 pathological labels. "Ignore" labels are assigned to labels when NLP cannot classify the label if there are any conflicting terms in the second stage. The tables show labels' information for the training, validation, and test datasets which were exclusively divided by patients.

AP view	(Gender) Male : Female = 58 % : 42 %, (Age) Average | Std. = 62 | 18															
	Train dataset						Validation dataset						Test dataset			
	Positive		Ignore		Negative		Positive		Ignore		Negative		Positive		Negative	
Fracture	3,446	4.8%	521	0.7%	68,171	94.5%	576	4.1%	89	0.6%	13,364	95.3%	12	1.2%	988	98.8%
Non-fracture	1,275	1.8%	26	0.0%	70,837	98.2%	177	1.3%	6	0.0%	13,846	98.7%	5	0.5%	995	99.5%
Diaphragm	3,293	4.6%	116	0.2%	68,729	95.3%	580	4.1%	30	0.2%	13,419	95.7%	8	0.8%	992	99.2%
Foreign body	44,034	61.0%	2,487	3.4%	25,617	35.5%	9,866	70.3%	440	3.1%	3,723	26.5%	677	67.7%	323	32.3%
Aorta	4,638	6.4%	351	0.5%	67,149	93.1%	716	5.1%	51	0.4%	13,262	94.5%	63	6.3%	937	93.7%
Cardiomegaly	10,583	14.7%	1,904	2.6%	59,651	82.7%	2,171	15.5%	407	2.9%	11,451	81.6%	227	22.7%	773	77.3%
Hilar area	266	0.4%	205	0.3%	71,667	99.3%	39	0.3%	29	0.2%	13,961	99.5%	2	0.2%	998	99.8%
Mediastinum	1,423	2.0%	449	0.6%	70,266	97.4%	255	1.8%	88	0.6%	13,686	97.6%	16	1.6%	984	98.4%
Cavity/Cyst	325	0.5%	110	0.2%	71,703	99.4%	116	0.8%	18	0.1%	13,895	99.0%	6	0.6%	994	99.4%
Emphysema	982	1.4%	2,261	3.1%	68,895	95.5%	170	1.2%	480	3.4%	13,379	95.4%	2	0.2%	998	99.8%
Atelectasis	36,785	51.0%	407	0.6%	34,946	48.4%	7,734	55.1%	126	0.9%	6,169	44.0%	554	55.4%	446	44.6%
Nodule/mass	3,663	5.1%	388	0.5%	68,087	94.4%	575	4.1%	49	0.3%	13,405	95.6%	18	1.8%	982	98.2%
Other interstitial opacity	2,334	3.2%	102	0.1%	69,702	96.6%	452	3.2%	20	0.1%	13,557	96.6%	8	0.8%	992	99.2%
Pulmonary edema	18,620	25.8%	2,921	4.0%	50,597	70.1%	4,568	32.6%	504	3.6%	8,957	63.8%	276	27.6%	724	72.4%
Pneumonia	19,581	27.1%	3,209	4.4%	49,348	68.4%	4,322	30.8%	579	4.1%	9,128	65.1%	174	17.4%	826	82.6%
Decreased lung volume	17,539	24.3%	879	1.2%	53,720	74.5%	3,352	23.9%	187	1.3%	10,490	74.8%	174	17.4%	826	82.6%
Increased lung volume	820	1.1%	16	0.0%	71,302	98.8%	158	1.1%	4	0.0%	13,867	98.8%	0	0.0%	1,000	100%
Other pleural lesions	1,678	2.3%	1,942	2.7%	68,518	95.0%	329	2.3%	348	2.5%	13,352	95.2%	4	0.4%	996	99.6%
Pleural effusion	23,020	31.9%	263	0.4%	48,855	67.7%	5,759	41.1%	51	0.4%	8,219	58.6%	348	34.8%	652	65.2%
Pneumothorax	5,485	7.6%	1,612	2.2%	65,041	90.2%	1,361	9.7%	351	2.5%	12,317	87.8%	71	7.1%	929	92.9%
PA view	(Gender) Male : Female = 50 % : 50 %, (Age) Average | Std. = 57 | 19															
	Train dataset						Validation dataset						Test dataset			
	Positive		Ignore		Negative		Positive		Ignore		Negative		Positive		Negative	
Fracture	1,919	2.9%	474	0.7%	64,155	96.4%	372	3.0%	97	0.8%	11,785	96.2%	31	3.1%	969	96.9%
Non-fracture	2,787	4.2%	25	0.0%	63,736	95.8%	524	4.3%	7	0.1%	11,723	95.7%	10	1.0%	990	99.0%
Diaphragm	2,171	3.3%	132	0.2%	64,245	96.5%	393	3.2%	19	0.2%	11,842	96.6%	15	1.5%	985	98.5%
Foreign body	16,011	24.1%	1,189	1.8%	49,348	74.2%	3,208	26.2%	191	1.6%	8,855	72.3%	244	24.4%	756	75.6%
Aorta	3,272	4.9%	403	0.6%	62,873	94.5%	609	5.0%	85	0.7%	11,560	94.3%	167	16.7%	833	83.3%
Cardiomegaly	4,289	6.4%	1,001	1.5%	61,258	92.1%	805	6.6%	170	1.4%	11,279	92.0%	104	10.4%	896	89.6%
Hilar area	197	0.3%	97	0.1%	66,254	99.6%	37	0.3%	18	0.1%	12,199	99.6%	11	1.1%	989	98.9%
Mediastinum	794	1.2%	388	0.6%	65,366	98.2%	121	1.0%	87	0.7%	12,046	98.3%	8	0.8%	992	99.2%
Cavity/Cyst	221	0.3%	78	0.1%	66,249	99.6%	38	0.3%	19	0.2%	12,197	99.5%	3	0.3%	997	99.7%
Emphysema	830	1.2%	337	0.5%	65,381	98.2%	159	1.3%	61	0.5%	12,034	98.2%	2	0.2%	998	99.8%
Atelectasis	12,905	19.4%	245	0.4%	53,398	80.2%	2,539	20.7%	40	0.3%	9,675	79.0%	235	23.5%	765	76.5%
Nodule/mass	3,981	6.0%	503	0.8%	62,064	93.3%	733	6.0%	92	0.8%	11,429	93.3%	36	3.6%	964	96.4%
Other interstitial opacity	4,047	6.1%	209	0.3%	62,292	93.6%	780	6.4%	37	0.3%	11,437	93.3%	12	1.2%	988	98.8%
Pulmonary edema	2,417	3.6%	4,121	6.2%	60,010	90.2%	492	4.0%	783	6.4%	10,979	89.6%	35	3.5%	965	96.5%
Pneumonia	8,602	12.9%	4,836	7.3%	53,110	79.8%	1,663	13.6%	939	7.7%	9,652	78.8%	123	12.3%	877	87.7%
Decreased lung volume	4,530	6.8%	274	0.4%	61,744	92.8%	833	6.8%	50	0.4%	11,371	92.8%	23	2.3%	977	97.7%
Increased lung volume	2,290	3.4%	13	0.0%	64,245	96.5%	422	3.4%	4	0.0%	11,828	96.5%	8	0.8%	992	99.2%
Other pleural lesions	1,659	2.5%	1,372	2.1%	63,517	95.4%	370	3.0%	261	2.1%	11,623	94.9%	8	0.8%	992	99.2%
Pleural effusion	7,044	10.6%	299	0.4%	59,205	89.0%	1,512	12.3%	57	0.5%	10,685	87.2%	136	13.6%	864	86.4%
Pneumothorax	885	1.3%	388	0.6%	65,275	98.1%	155	1.3%	68	0.6%	12,031	98.2%	9	0.9%	991	99.1%

Supplementary Table 2 | Model performance. xAI model performance for 20 clinical output labels was evaluated on PA test datasets of our institution (PA view in Supplementary Table 1) based on both NLP and human labels. The 95% CIs on the metrics were provided in parentheses (Statistical analyses in Methods). Out of 20 clinical output labels, in this study, we selected 5 typical clinical output labels that were commonly defined in three public datasets.

<table>
<tr><th>Class</th><th>Test (NLP)</th><th>Test (Human)</th><th>Class</th><th>Test (NLP)</th><th>Test (Human)</th></tr>
<tr><td>Fracture</td><td>0.77 (0.67, 0.86)</td><td>0.85 (0.77, 0.91)</td><td>Nodule/mass</td><td>0.68 (0.60, 0.76)</td><td>0.80 (0.71, 0.88)</td></tr>
<tr><td>Non-fracture</td><td>0.91 (0.85, 0.96)</td><td>0.99 (0.98, 1.00)</td><td>Other interstitial opacity</td><td>0.83 (0.78, 0.87)</td><td>0.94 (0.89, 0.98)</td></tr>
<tr><td>Diaphragm</td><td>0.93 (0.90, 0.96)</td><td>0.97 (0.94, 0.99)</td><td>Pulmonary edema</td><td>0.95 (0.91, 0.97)</td><td>0.98 (0.97, 0.99)</td></tr>
<tr><td>Foreign body</td><td>0.92 (0.90, 0.94)</td><td>0.95 (0.93, 0.97)</td><td>Pneumonia</td><td>0.77 (0.73, 0.82)</td><td>0.90 (0.88, 0.93)</td></tr>
<tr><td>Aorta</td><td>0.91 (0.88, 0.94)</td><td>0.94 (0.92, 0.96)</td><td>Decreased lung volume</td><td>0.92 (0.89, 0.94)</td><td>0.98 (0.96, 0.99)</td></tr>
<tr><td>Cardiomegaly</td><td>0.93 (0.91, 0.95)</td><td>0.97 (0.95, 0.98)</td><td>Increased lung volume</td><td>0.91 (0.86, 0.95)</td><td>0.98 (0.96, 0.99)</td></tr>
<tr><td>Hilar area</td><td>0.82 (0.58, 0.98)</td><td>0.94 (0.90, 0.98)</td><td>Other pleural lesions</td><td>0.85 (0.76, 0.92)</td><td>0.98 (0.94, 1.00)</td></tr>
<tr><td>Mediastinum</td><td>0.92 (0.86, 0.97)</td><td>0.98 (0.95, 1.00)</td><td>Pleural effusion</td><td>0.97 (0.96, 0.98)</td><td>0.98 (0.97, 0.99)</td></tr>
<tr><td>Cavity/Cyst</td><td>0.87 (0.76, 0.95)</td><td>0.94 (0.87, 1.00)</td><td>Pneumothorax</td><td>0.89 (0.78, 0.97)</td><td>0.95 (0.91, 0.98)</td></tr>
<tr><td>Emphysema</td><td>0.94 (0.90, 0.98)</td><td>1.00 (0.99, 1.00)</td><td rowspan="2">Mean AUROC</td><td rowspan="2">0.88 (0.86, 0.89)</td><td rowspan="2">0.95 (0.94, 0.96)</td></tr>
<tr><td>Atelectasis</td><td>0.89 (0.86, 0.91)</td><td>0.94 (0.93, 0.96)</td></tr>
</table>

Supplementary Table 3 | Automated-labeling model CXR "capture rate" applied to the three complete public datasets, at the optimized pSim threshold value for maximal accuracy per clinical output label (PPV, NPV = 1; Figs. 2 - 4). Our automated-labeling model was applied to the three external open source CXR datasets: CheXpert, MIMIC, and NIH. Dataset composition and number of positively labelled, negatively labelled, and unlabelled PA CXR's for each of the five clinical output labels (cardiomegaly, pleural effusion, edema, pneumonia, and atelectasis) are shown (**Supplementary Table 3**, A). The minimal optimal pSim threshold value, such that PPV, NPV = 1 for labeling accuracy, was selected as per **Figs. 2 - 4 (Supplementary Table 3,** B**)**. It is noteworthy that the model's mean CXR "capture rates" for the pooled results from the three full public datasets (**Supplementary Table 3**, C), closely correspond to those shown in the graphs of **Figs. 2 - 4 b**, for the randomly selected subset of examinations (n=90-100) labelled by both the model and the expert radiologists.

(A) CheXpert (public labels)					(B) Model (auto-labels) for pSim value (PPV, NPV = 1)				
	Positive	Negative	Unlabelled	Total # PA CXR's	pSim	Positive	Negative	# CXR's Labelled	Capture rate (%)
Cardiomegaly	2,909	3,188	23,323	29,420	0.15	3,532	15,862	19,394	66
Pleural Effusion	8,078	9,583	11,759	29,420	0.20	4,490	13,517	18,007	61
Edema	1,709	4,357	23,354	29,420	0.55	32	5,776	5,808	20
Pneumonia	1,198	858	27,364	29,420	0.65	1,076	1,684	2,760	9
Atelectasis	3,195	271	25,954	29,420	0.60	3,150	3,654	6,804	23
(A) MIMIC (public labels)					(B) Model (auto-labels) for pSim value (PPV, NPV = 1)				
	Positive	Negative	Unlabelled	Total # PA CXR's	pSim	Positive	Negative	# CXR's Labelled	Capture rate (%)
Cardiomegaly	7,427	6,124	57,672	71,223	0.15	5,536	50,347	55,883	78
Pleural Effusion	9,275	10,312	51,636	71,223	0.20	5,987	42,240	48,227	68
Edema	2,032	9,084	60,107	71,223	0.55	30	19,585	19,615	28
Pneumonia	4,793	11,660	54,770	71,223	0.65	2,081	10,980	13,061	18
Atelectasis	7,328	376	63,519	71,223	0.60	4,052	14,960	19,012	27
(A) NIH (public labels)					(B) Model (auto-labels) for pSim value (PPV, NPV = 1)				
	Positive	Negative	Unlabelled	Total # PA CXR's	pSim	Positive	Negative	# CXR's Labelled	Capture rate (%)
Cardiomegaly	1,563	65,747	0	67,310	0.15	875	57,924	58,799	87
Pleural Effusion	6,589	60,721	0	67,310	0.20	7,615	40,381	47,996	71
Edema	276	67,034	0	67,310	0.55	93	20,144	20,237	30
Pneumonia	630	66,680	0	67,310	0.65	1,828	15,659	17,487	26
Atelectasis	5,728	61,582	0	67,310	0.60	3,094	18,526	21,620	32

(C) Pooled model labels for the 3 public datasets			
	Total # PA CXR's	# CXR's Labelled	Weighted Capture Rate (%)
Cardiomegaly	167,953	134,076	80
Pleural Effusion	167,953	114,230	68
Edema	167,953	45,660	27
Pneumonia	167,953	33,308	20
Atelectasis	167,953	47,436	28

Supplementary Table 4 | Stratified performance of the six DenseNet-121 models that form the ensemble for each of the original and fine-tuned models. To demonstrate the ability of our system to generalize to external datasets at a user designated level of performance, we fine-tuned our original model through iterative re-training using the auto-labelled CXR exams from the three public datasets. The CXR exams selected for re-training (n=31,020) had at least one positive label, a pSim value greater than or equal to the optimal threshold for that label (as per Figs. 2-4c, 2-4d, and 5), and were excluded if they had been used previously as part of the test set. The ensemble and DenseNet-121 component performance of the original model, compared to the fine-tuned model, for each of the five model labels and their average, are shown. The fine-tuned model utilized the same environments and hyper-parameters (e.g., learning rate=10^{-8}) as the original model. Performance for each of the model labels was preserved or improved on the more generalized, fine-tuned model.

Original Models							
	Model 1	Model 2	Model 3	Model 4	Model 5	Model 6	Ensemble
Cardiomegaly	0.972	0.983	0.976	0.998	0.997	1.000	0.994
Pleural Effusion	0.993	0.985	0.984	0.997	0.996	0.996	0.998
Edema	0.949	0.930	0.933	0.968	0.956	0.967	0.960
Pneumonia	0.905	0.818	0.869	0.925	0.884	0.912	0.908
Atelectasis	0.932	0.946	0.936	0.949	0.928	0.957	0.954
Average Score	0.950	0.932	0.940	0.967	0.952	0.966	0.963
Finetuned Models							
	Model 1	Model 2	Model 3	Model 4	Model 5	Model 6	Ensemble
Cardiomegaly	0.972	0.986	0.988	0.997	0.998	0.998	0.998
Pleural Effusion	0.994	0.994	0.983	0.998	0.998	0.998	0.998
Edema	0.977	0.919	0.954	0.967	0.964	0.971	0.968
Pneumonia	0.923	0.870	0.892	0.933	0.894	0.918	0.930
Atelectasis	0.942	0.974	0.948	0.960	0.946	0.963	0.965
Average Score	0.961	0.949	0.953	0.971	0.960	0.970	0.972

Supplementary Table 5 | Comparison of model performance when training using different combinations of PA and AP CXR's. To assess the impact on model performance by training using PA CXR's only, we compared performance when training using various combinations of PA and AP CXR's for the weighted loss function (W = 1.1, 1.5, and 2.0). The AUROC performance showed minimal differences between using PA CXR's only and three different weightings. This result is in keeping with our clinical experience of CXR interpretation by human experts.

Class	Only PA CXRs	PA + AP CXRs		
Hyper-parameter, w	-	w=1.1	w=1.5	w=2.0
Fracture	0.778	0.790	0.810	0.806
Non-fracture	0.855	0.849	0.856	0.865
Diaphragm	0.897	0.897	0.888	0.899
Foreign body	0.888	0.892	0.895	0.894
Aorta	0.884	0.883	0.881	0.880
Cardiomegaly	0.922	0.920	0.919	0.921
Hilar area	0.883	0.924	0.895	0.927
Mediastinum	0.927	0.944	0.927	0.930
Cavity/Cyst	0.814	0.853	0.854	0.845
Emphysema	0.921	0.921	0.919	0.929
Atelectasis	0.861	0.868	0.866	0.864
Nodule/mass	0.715	0.718	0.720	0.714
Other interstitial opacity	0.793	0.803	0.798	0.798
Pulmonary edema	0.932	0.933	0.932	0.933
Pneumonia	0.789	0.802	0.800	0.800
Decreased lung volume	0.882	0.881	0.883	0.883
Increased lung volume	0.896	0.891	0.885	0.892
Other pleural lesions	0.847	0.852	0.848	0.858
Pleural effusion	0.965	0.967	0.964	0.966
Pneumothorax	0.882	0.884	0.890	0.895
Mean AUROC	0.867	0.874	0.871	0.875

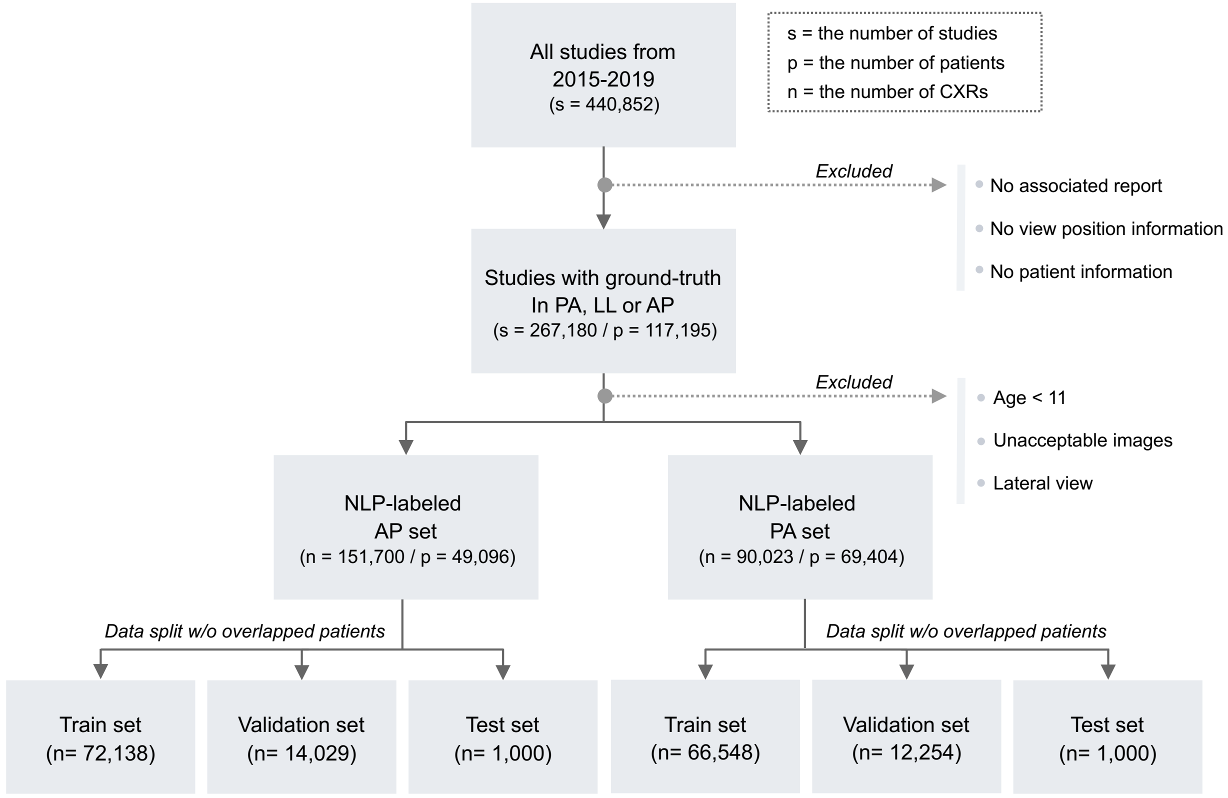

Supplementary Figure 1 | CXR data collection for the xAI model development. Each dataset for AP and PA view positions collected and mapped one-on-one to the annotations extracted by our NLP tool from the corresponding radiological reports. For each view position, training, validation, and test datasets were divided without overlapped patients or duplicated cases.

a. Cardiomegaly: 39/50 (78%) positive cases with complete agreement

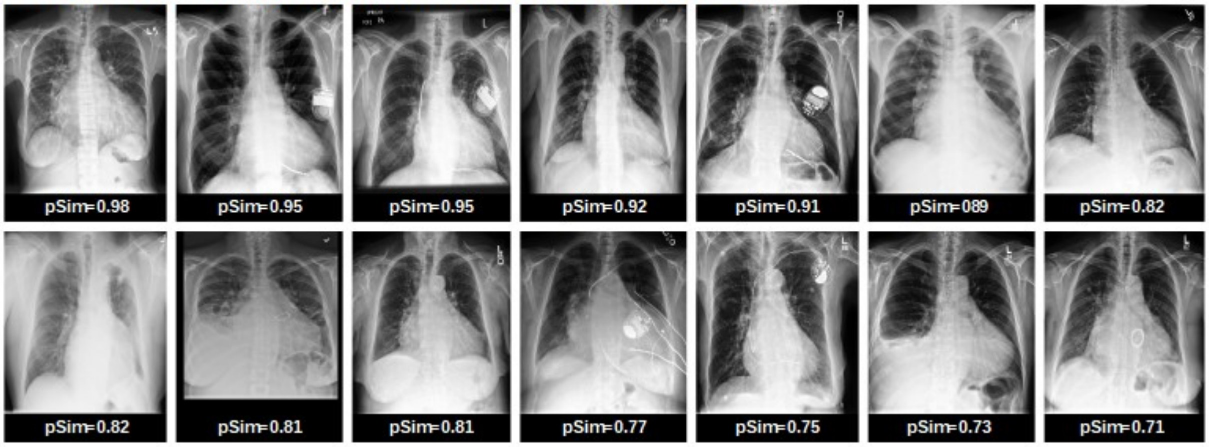

b. Pleural Effusion: 39/50 (78%) positive cases with complete agreement

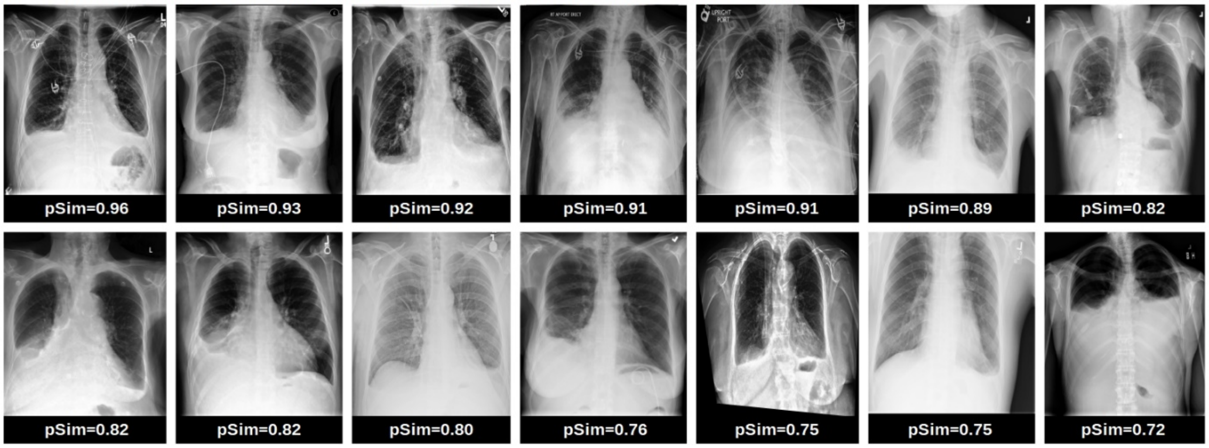

c. Pulmonary Edema: 17/40 (43%) positive cases with complete agreement

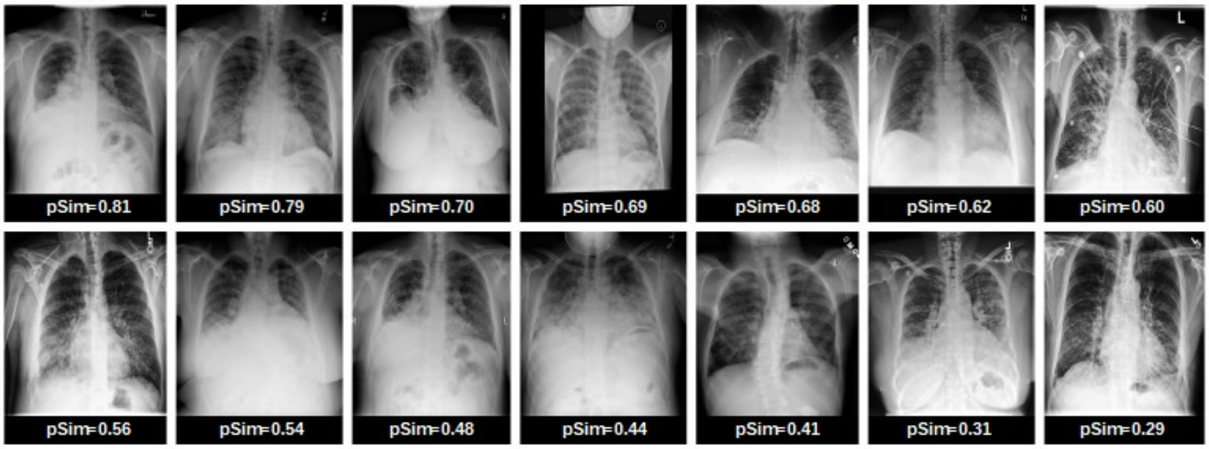

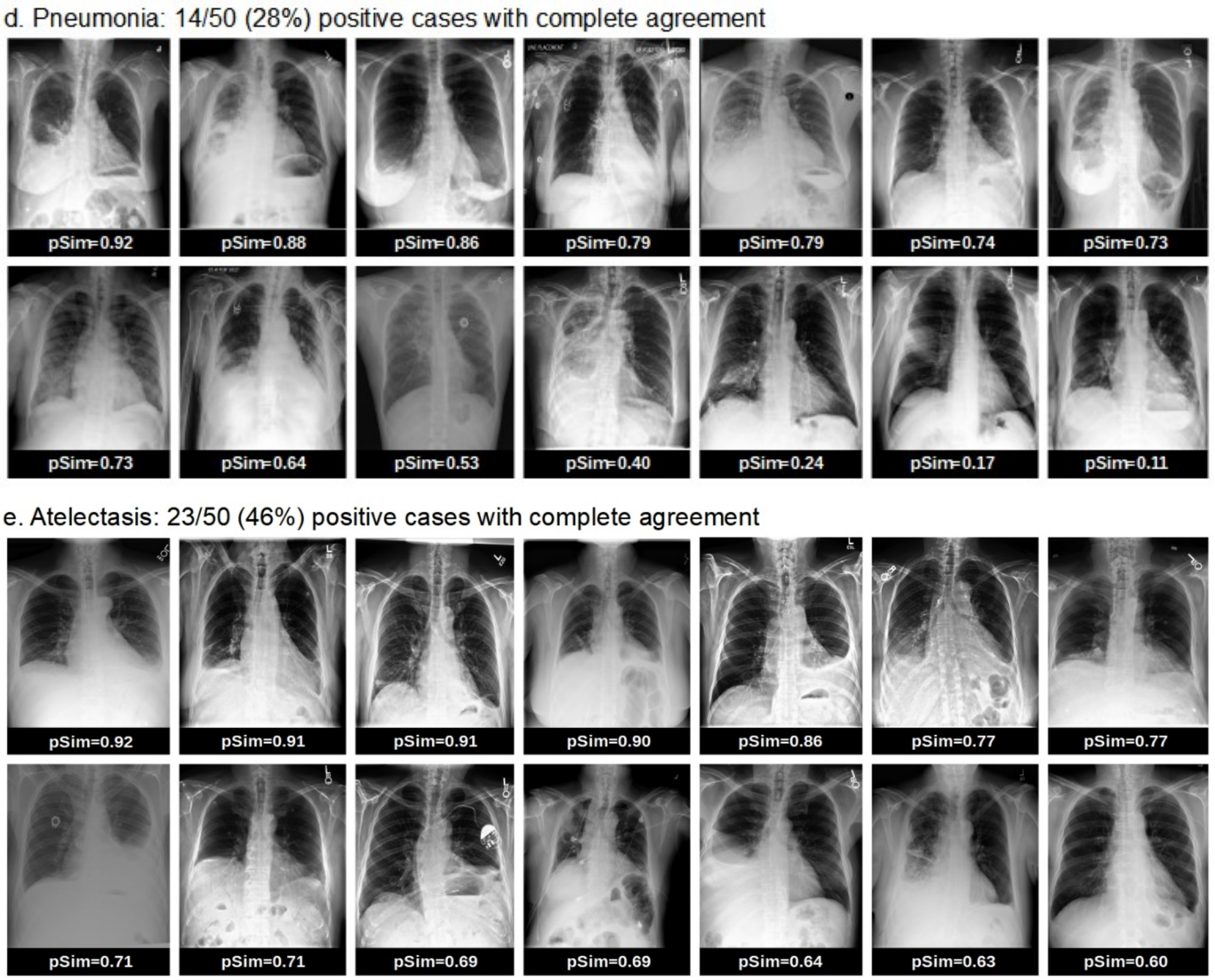

Supplementary Figure 2 | Sample PA CXR images of positive examinations identified by the automated-labeling model, which had complete agreement between all 7 expert radiologists and the xAI model output, for each of the five clinical output labels studied. Shown are fourteen sample PA CXR images with positive findings, for each of the five clinical output labels studied (cardiomegaly **a**, pleural effusion **b**, pulmonary edema **c**, pneumonia **d**, and atelectasis **e**), for which there was complete agreement between both the xAI model output and the 7 expert radiologist interpretations. The pSim threshold values used by the model for auto-labeling clinical output label detection are shown (**a-e**). For pneumonia, **d**, there were only 14 positive examinations, out of 50 total positive examinations identified by the model, which had complete agreement with all readers (14/50=28%), as per **Figs. 2 - 4, a**; percent agreement for the other clinical output labels is as shown in the above captions (**a-c, e**).

Ensemble performance (AUROC)	DenseNet-121	ResNet-50	MobileNet v2	MnasNet
**Category**	**Testset (Annotation)**	**Testset (Annotation)**	**Testset (Annotation)**	**Testset (Annotation)**
Cardiomegaly	0.97	0.97	0.98	0.97
Pleural effusion	0.98	0.99	0.98	0.98
Pulmonary edema	0.98	0.97	0.98	0.98
pneumonia	0.90	0.90	0.90	0.91
Atelectasis	0.94	0.94	0.94	0.94
**mean AUROC**	**0.95**	**0.95**	**0.96**	**0.96**

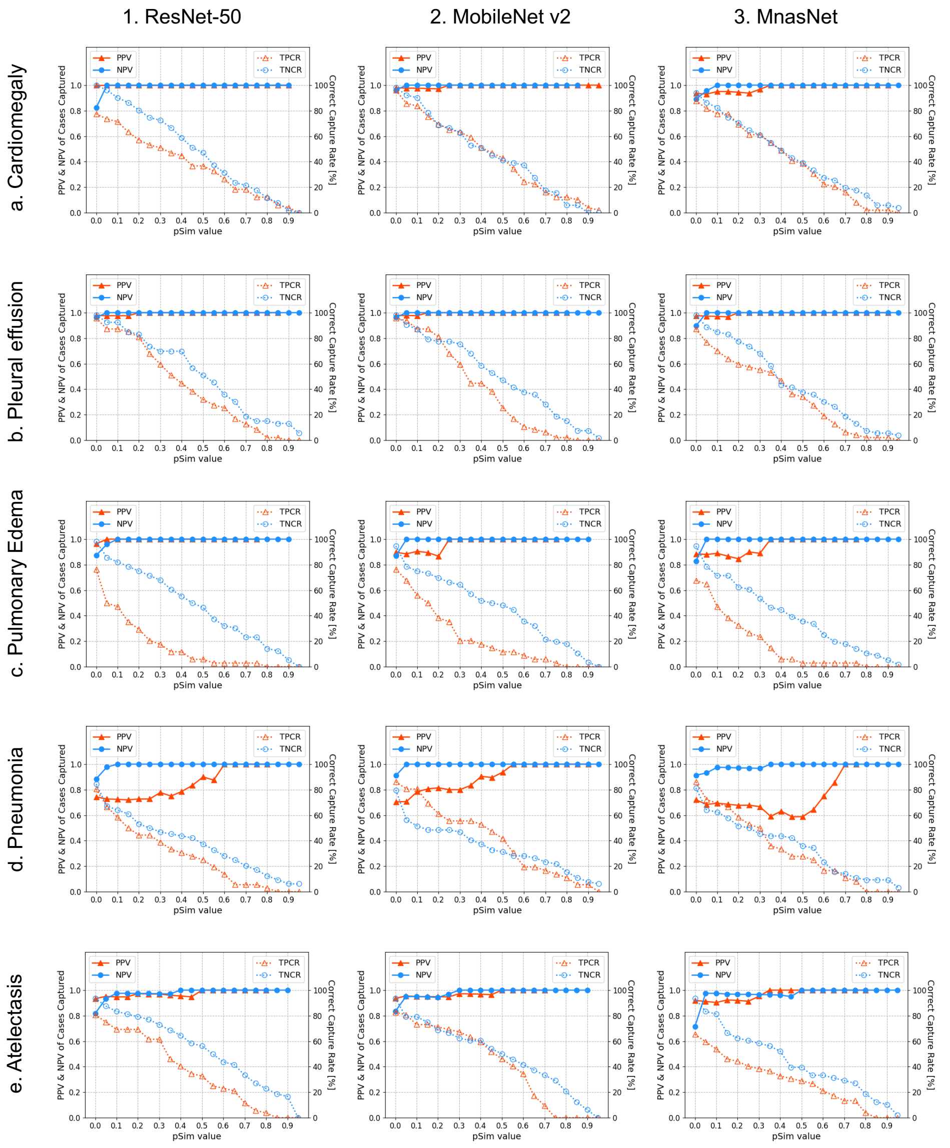

Supplementary Figure 3 | Performance comparison between three additional, different model architectures: RestNet 50, MobileNet v2, and MnasNet. We replicated the analysis shown in Figs. 2-4 by retraining three new models with the following three architectures, ResNet-50 [13], MobileNet v2 [14], and MnasNet [15], using the same training/validation/test datasets, parameters, and environments as for our primary DenseNet-121 model (**Methods**). Our results show consistent performance, similar to that of Figs. 2-4 for all three new models.

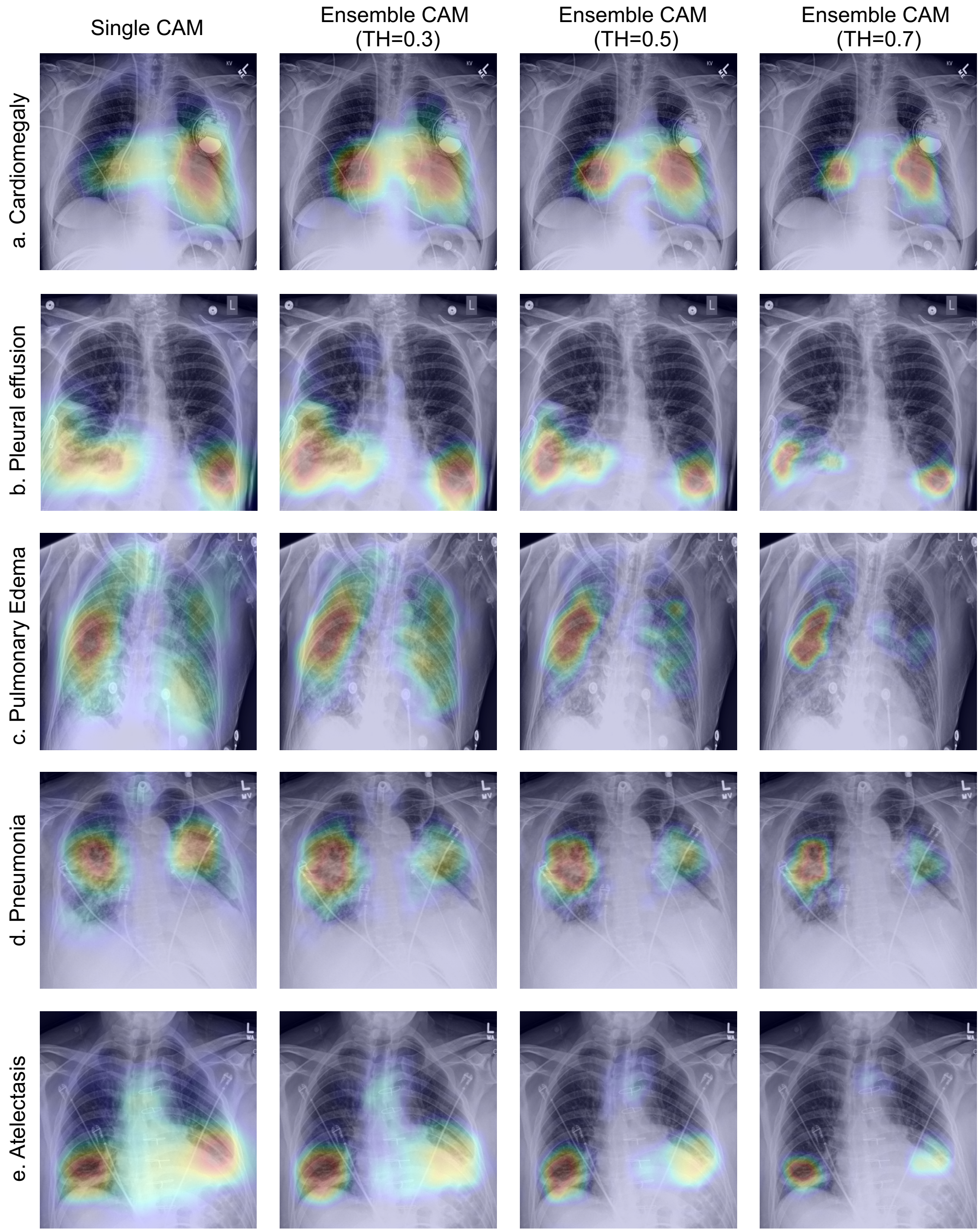

Supplementary Figure 4 | Representative impact of varying ensemble CAM method thresholds on attention map visualization. We considered three thresholds (0.3, 0.5, and 0.7, columns 2-4) for the ensemble CAM method (**Methods**), compared to a single CAM method without noise reduction (column 1), for each of the 5 different clinical output labels. Visual review of several such examples from each category by three human experts (**Methods**) suggested that an ensemble CAM threshold of 0.5 optimally correlated with the reference standard pathology present on the CXR source images.

Reference

[1] Langlotz, C. P. *RadLex: a new method for indexing online educational materials.* http://radlex.org/ (2006).

[2] Bird, S., Klein, E. and Loper, E. *Natural language processing with Python: analyzing text with the natural language toolkit.* (O'Reilly Media, Inc., 2009).

[3] Honnibal, M., & Montani, I. spacy 2: Natural language understanding with bloom embeddings. *convolutional neural networks and incremental parsing*, **7(1)**, (2017).
